# Genetic diversity and virulence of *Bacillus cereus* group isolates from bloodstream infections

**DOI:** 10.1128/spectrum.02407-24

**Published:** 2025-01-28

**Authors:** Akiko Okutani, Shu Okugawa, Fumie Fujimoto, Mahoko Ikeda, Takeya Tsutsumi, Kyoji Moriya, Ken Maeda

**Affiliations:** 1 Department of Veterinary Science, National Institute of Infectious Diseases, Tokyo, Japan; 2 Department of Infectious Diseases, The University of Tokyo Hospital, Tokyo, Japan; 3 Department of Infection Control and Prevention, The University of Tokyo Hospital, Tokyo, Japan; 4 Tokyo Healthcare University, Tokyo, Japan; bioMerieux Inc, Denver, Colorado, USA

**Keywords:** *Bacillus cereus *group, bloodstream infection, whole-genome analysis, epidemiology

## Abstract

*Bacillus cereus* catheter-related bloodstream infections (CRBSIs) are an increasing concern in Japanese hospitals. Although their clinical characteristics have been explored, the genetic relationships and virulence profiles of *B. cereus* isolates from CRBSIs remain understudied. Here, using advanced genomic techniques, we investigated the genetic diversity, phylogenetic relationships, and virulence profiles of *B. cereus* isolates from patients with bloodstream infections. We analyzed 28 *B. cereus* group strains isolated from blood samples at the University of Tokyo Hospital between 2005 and 2017 using whole-genome sequencing, core-genome single-nucleotide polymorphism (SNP) typing, and virulence gene profiling. Core-genome SNP analysis revealed significant genetic diversity among the isolates, suggesting multiple independent sources of infection. The isolates predominantly belonged to *panC* clades III and IV, with distinct virulence gene profiles. All *panC* clade III isolates contained *hbl* operon genes, whereas four isolates from clade IV harbored cereulide synthetase genes (*cesABCD*). One isolate possessed a capsule gene operon (*capBCADE*), a rare finding among clinical *B. cereus* strains. Biofilm formation ability was observed in 50% of catheter-related isolates, although this ability was not significantly different from that of the noncatheter-related isolates.

**IMPORTANCE:**

This study provides novel insights into the genetic diversity and virulence potential of *B. cereus* strains causing bloodstream infections in a Japanese hospital setting. These findings suggest diverse infection pathways and highlight the importance of continuous molecular epidemiological surveillance for effective infection control.

## INTRODUCTION

In nature, *Bacillus cereus* mainly inhabits soil, water systems, and the intestinal tracts of animals and humans. It is not generally pathogenic but can cause food poisoning and sepsis (1–3). *B. cereus* group strains usually possess endogenous β-lactamases, such as zinc metallo-β-lactamases, and therefore harbor intrinsic resistance to β-lactam antibiotics. As a consequence, vancomycin is the first-line drug of choice to treat *B. cereus* infections (4). Several outbreaks of *B. cereus* catheter-related bloodstream infection (CRBSI) have been reported in hospitals in Japan (5–9). In one outbreak, *B. cereus* was transmitted via catheters from contaminated hospital linens to patients (8). Pulsed-field gel electrophoresis and multilocus sequence typing (MLST) revealed that *B. cereus* sequence type 1420 was the dominant sequence type isolated from patients at three locations in Japan with nosocomial infections diagnosed in 2006, 2013, and 2016 (9). Ikeda et al. (10) reported on the clinical characteristics and antimicrobial susceptibility of 29 patients with *B. cereus* bloodstream infections (BSIs) at the University of Tokyo Hospital. The most common source of CRBSI was venous catheters, accounting for 69% of cases. Although the 4-week mortality rate does not differ significantly according to whether patients are treated with appropriate or inappropriate empirical antimicrobial therapy, patients treated with appropriate antimicrobial therapy are significantly more likely to experience defervescence within 2 days (10). However, the genotypes and presence of virulence genes were not examined in this study.

Recently, disease outbreaks caused by pathogenic strains of *B. cereus* with virulence characteristics similar to those of *B. anthracis* have been reported in humans and animals in various locations worldwide (11–13). These *B. cereus* strains possess anthrax toxins, protective antigens, lethal factors, and edematous factors and can cause severe life-threatening toxemia (13). They also express a capsule that surrounds bacterial cells in the host blood and aids the evasion of host immune cells. Although *B. cereus* strains harboring anthrax virulence factors have not been reported in hospital CRBSIs, ongoing vigilance is required.

A previous study assessed the genetic relatedness of *B. cereus* isolates from different origins, including the environment, animal feces, and human clinical samples, to assess the genetic diversity and characteristics of *B. cereus* in Japan (14). The soil- and animal fecal-derived strains formed closely related clusters, but the nosocomial infection strains were classified into different clusters. Some nosocomial infections and animal-derived strains possess a partial capsular gene operon (14, 15).

The present study focused on the genetic relationships and the phylogenetic parameters of *B. cereus* BSIs using core-genome single-nucleotide polymorphism (SNP) typing of the whole-genome sequence. We also investigated biofilm formation ability and the genetic profile of virulence-related genes.

## MATERIALS AND METHODS

### Bacterial isolates

*B. cereus* group isolates were isolated from the blood of patients hospitalized at the University of Tokyo Hospital between 2005 and 2017, and the isolates were detected in two or more sets of blood cultures, as reported previously (10). Data regarding age, sex, the day of positive blood culture, and the source of bacteremia were extracted from the medical records. CRBSI was determined when (i) *B. cereus* grew in a catheter tip culture and percutaneously drawn blood cultures, (ii) there were local signs of phlebitis and the absence of other evident infectious foci, or (iii) *B. cereus* was not related to an infection at another site, and the patient was clinically diagnosed with CRBSI. Other or unknown sources of bacteremia were classified as non-CRBSIs. Ethical approval for the study was obtained from the University of Tokyo Hospital (2020, No. 2019342NI) and the National Institute of Infectious Diseases (2020, No. 1130). The requirement for informed consent was waived owing to the retrospective design.

Bacterial DNA was extracted as reported previously (15). Briefly, blood was added to BACTEC standard culture bottles, which were then incubated in a BACTEC 9000 system (Becton Dickinson and Company, Franklin Lakes, NJ, USA) at 37°C for 24 h. All of the isolates were identified as *B. cereus* using the VITEK2 system with a BCL identification card (Sysmex bioMérieux, Tokyo, Japan) and matrix-assisted laser desorption/ionization (MALDI) time-of-flight mass spectrometry, generating a mass spectrum pattern of *B. cereus* using a MALDI Biotyper (version 2.0) (Bruker Daltonics, Bremen, Germany), in accordance with the manufacturer’s instructions. The isolates were stored as frozen stocks. Cultures from frozen stocks were incubated in Luria–Bertani broth at 37°C for 24 h. Genomic DNA was extracted from the broth culture using a QIAamp DNA Mini Kit (Qiagen, Hilden, Germany).

### Measurement of biofilm formation

Biofilm formation was measured using a Biofilm Formation Assay Kit (Dojindo, Tokyo, Japan) according to the manufacturer’s instructions. Briefly, 180 µL of microbial cell suspension in biofilm-inducing minimal salts glycerol glutamate (MSgg) medium (15) was added to each well of a 96-well plate. A 96-peg lid was placed on the plate, which was incubated at 37°C to allow biofilm to form on the pegs. The 96-peg lid was washed by soaking in sterile physiological saline. The lid was then placed in 1 vol% crystal violet solution in a fresh 96-well plate and incubated at room temperature for 30 min. The lid was washed again by soaking in physiological saline solution and then soaked in 99.5 vol% ethanol solution in a fresh 96-well plate at room temperature for 15 min. The 96-peg lid was removed, and the absorbance of each well at 590 nm was measured using a microplate reader (iMark; Bio-Rad Japan, Tokyo, Japan). The absorbance of blank cells was used as a biofilm-negative control. The cutoff value (ODc) in each plate was defined as three standard deviations (SD) above the mean OD of the negative control: ODc = average OD of negative control + (3 × SD of negative control). Interpretation of results was performed after calculation of the average ODs of all strains, according to the published criteria (16). The experiments were independently performed three times using three replicate samples each time. Biofilm formation by different isolates was compared using a one-way analysis of variance followed by a Dunnett test for multiple comparisons using GraphPad Prism software version 9.3.1 (GraphPad Software, San Diego, CA, USA).

### Whole-genome sequencing and phylogeny using core-genome single-nucleotide polymorphisms and whole-genome multilocus sequence typing

Genomic DNA libraries were prepared for each isolate using the NEBNext DNA Library Prep Master Mix Set for Illumina (New England Biolabs (NEB), Ipswich, MA, USA) and NEBNext Multiplex Oligos for Illumina (Index Primers Set one and Set 2; NEB) according to the manufacturer’s instructions. The libraries were then used for 2 × 151, 250, or 300 bp paired-end sequencing using the Illumina MiSeq platform (Illumina, San Diego, CA, USA) with a MiSeq Reagent Kit v2 (300 cycles, 500 cycles) or v3 (600 cycles). After filtering low-quality reads and quality trimming using CLC Genomics Workbench 20 (Qiagen) with default parameters, standard settings were used to conduct *de novo* assembly of high-quality paired-end reads.

The Parsnp tool from Harvest Suite software was used for core-genome SNP typing, using the *B. cereus* ATCC 14579 chromosome (NC_004722.1) as the reference genome (16). The whole-genome data for the isolates from other countries are listed in the Supplemental table. The assembled contigs were used as inputs for Parsnp v1.2 using the parameters -c and -C 1000. The detected SNPs were extracted into a VCF file using HarvestTools v1.1.2, and the phylogeny was visualized using gingr (17).

Members of the *B. cereus* group can be classified into seven phylogenetic groups using *panC* gene sequencing (18). The “mapped reads to reference” option of the CLC Genomics Workbench was used to determine the total read count and average query coverage of *panC* sequences from clades I to VII, and the CLC Genomics Workbench was also used to detect virulence-associated genes from the BTyper3 program (19, 20), including hemolysin BL (*hbl*) operon genes (*hblABCD*), nonhemolytic enterotoxin operon genes (*nheABC*), cereulide synthetase genes (*cesABCD*), capsule biosynthesis operon genes (*capABCDE*), anthrax toxin genes (*cya, lef*, *pagA*), hemolysin genes (*hlyA*, *hlyI*, *hlyII*, and *hlyIII*), and a transcriptional regulator gene (*plcR*) and seven genes (*glp*, *gmk*, *ilv*, *pta*, *pur*, *pyc*, and *tpi*) for MLST. Each sequence type was determined using the seven gene sequences according to public databases for molecular typing and microbial genome diversity (PubMLST) for *Bacillus cereus* (https://pubmlst.org/organisms/bacillus-cereus).

## RESULTS

### Epidemiology and biofilm formation

**Fig 1:**
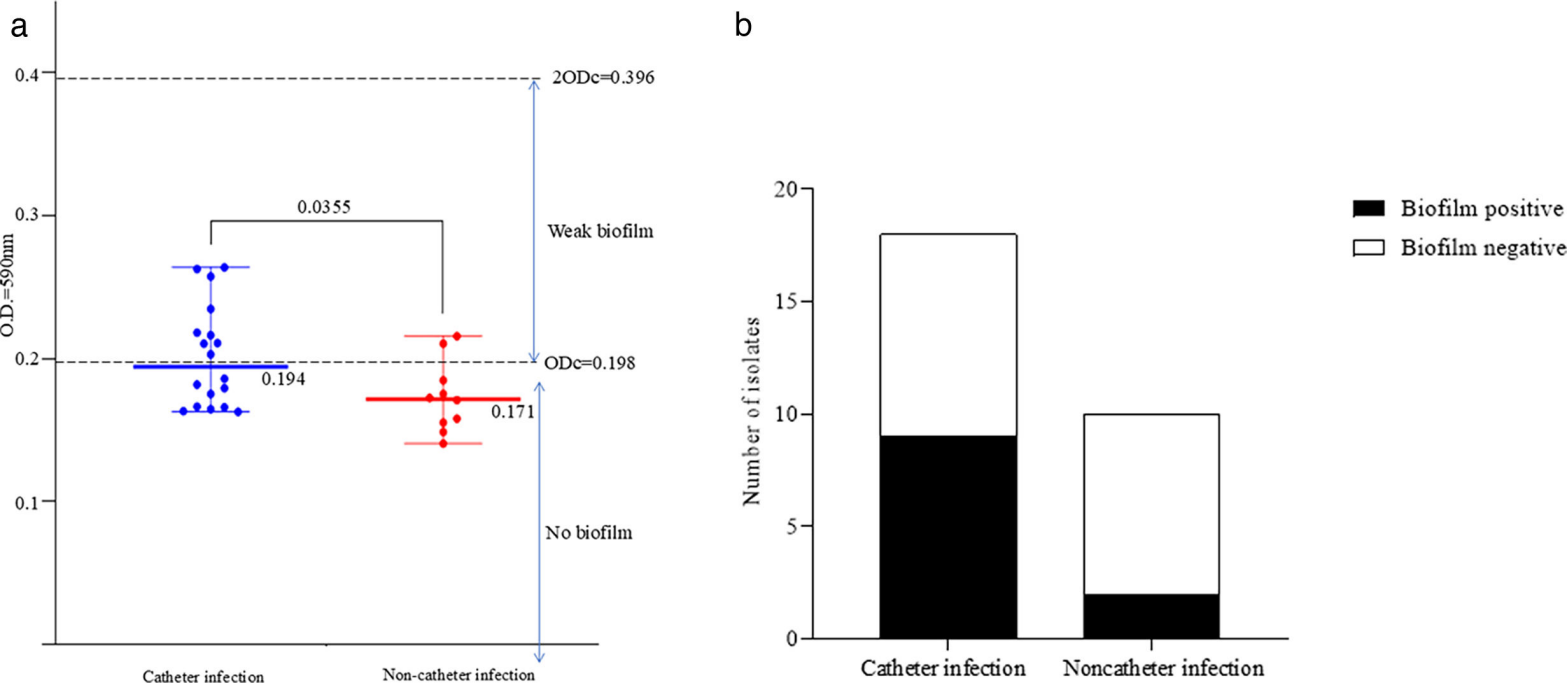
(**a**) The distribution of biofilm formation among the catheter and noncatheter groups showing the OD_590 nm_ values. ODc = Average OD of the negative control + 3 × SD of the negative control. Each dot represents the average OD value of each strain from triplicate samples in three independent experiments. One-way analysis of variance followed by a Dunnett test revealed a significant difference (*P* = 0.0355). The average value and range are shown for both groups. (**b**) Distribution of biofilm-forming ability among *B. cereus* isolates from catheter-related and noncatheter-related infections. The bar graph shows the number of *B. cereus* isolates that were biofilm-positive (black) or biofilm-negative (white) for catheter infection (*n* = 18) and noncatheter infection (*n* = 10) cases. Among the catheter infection isolates, nine (50%) were biofilm-positive; among the noncatheter infection isolates, two (20%) were biofilm-positive. However, this difference between infection source and biofilm formation ability was not statistically significant (*P* = 0.22, Fisher’s exact test).

Twenty-eight *B. cereus* isolates were analyzed in this study. The patients had a mean age of 57 years (range: 10–89 years), and 50% (14/28) were male. Detection was highest in the summer (from July to September; 50%, 14/28) and in patients aged 60–79 years (58%, 16/28). The probable source of infection was a venous catheter in 64% (18/28) of the patients, including 13 peripherally venous catheters and five central venous catheters. The distribution of biofilm formation among the catheter and noncatheter infection groups based on individual OD values is shown in Fig. 1a. A significant difference (*P* = 0.0355) in the OD values between the catheter and noncatheter groups was detected.

Nine of the 18 (50%) catheter-related isolates exhibited biofilm formation (Fig. 1b). The route of infection was via peripherally venous catheters (seven isolates) and central venous catheters (two isolates). Two of the 10 (20%) noncatheter-related isolates formed biofilms. However, statistical analysis did not reveal a significant association between infection source and biofilm formation ability (16) (*P* = 0.22, Fisher’s exact test).

### Whole-genome sequencing and phylogenetic analysis of the *panC* clade, MLST, and core-genome SNP

**Fig 2:**
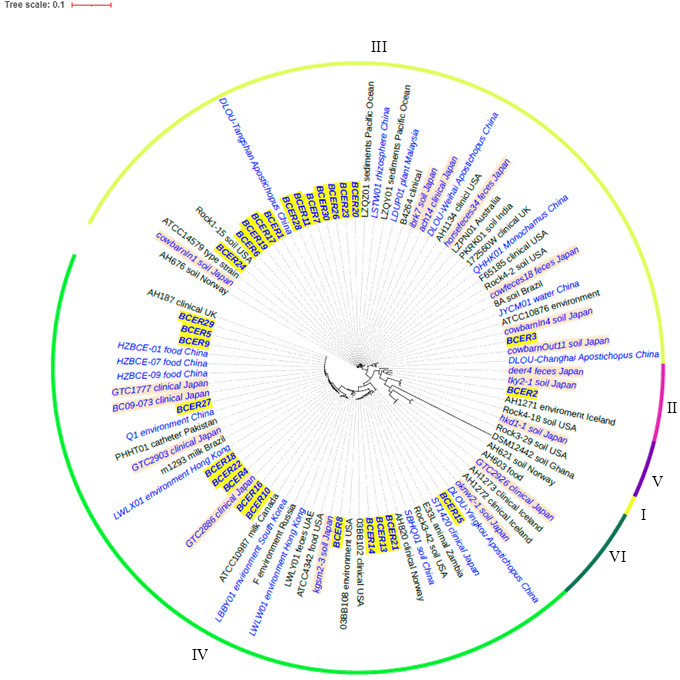
Phylogenetic tree of *B. cereus* isolates based on core-genome single-nucleotide polymorphism (SNP) analysis The circular phylogenetic tree represents the genetic relationships between *B. cereus* group isolates, which were classified into seven phylogenetic groups based on *panC* gene sequencing. The tree is color-coded to indicate *panC* clades (I–VI), as shown by the outer ring. Isolates are labeled and color-coded as follows: bold italic blue text with dark red highlighting: 28 isolates from the current study; italic blue text with light red highlighting: previously reported isolates from Japan; italic blue text without highlighting, isolates from other East Asian countries; and black text, isolates from other regions or reference strains. The tree scale of 0.1 indicates the genetic distance.

The results of MLST of the seven genes considered in this study are shown in Table 1. MLST analysis revealed diverse sequence types among the isolates. Notably, we did not identify any isolates belonging to ST1420, which has been previously reported to be prevalent in nosocomial *B. cereus* infections in Japan (9). The isolate most closely related to ST1420 was BCER15, which differed in the *glp* and *ilv* loci. Analysis of virulence-associated genes and *panC* genes using CLC Genomics Workbench revealed that 1, 14, and 13 isolates belonged to *panC* clades II, III, and IV, respectively (Table 1). Genomic data of the *B. cereus* group isolates from Japan and other countries revealed that previously isolated *B. cereus* group isolates of various origins also belonged predominantly to *panC* clades III and IV (Fig. 2).

**TABLE 1:** List of isolates and corresponding metadata^*a*^

Isolate name	Isolation year	Isolation month	MLST sequence type	glp	gmk	ilv	pta	pur	pyc	tpi	*panC* clade	Accession number	Average OD_450_ value	ANI by GTDB taxonomy (>95%) using DFAST ()
BCER1	2005	March	3281	13	8	9	14	9	12	4	IV	DRA017100	0.140	*B. cereus sensu stricto*
BCER2	2006	July	3282	3	2	31	5	11	3	4	II	DRR544813	0.182	*B. mobilis*
BCER3	2006	October	1009	16	6	170	9	4	7	21	IV	DRR544814	0.186	*B. cereus sensu stricto*
BCER4	2006	November	3283	19	2	21	47	114	3	120	III	DRR544815	0.235	*B. paranthracis*
BCER5	2007	January	26	3	2	31	5	16	3	4	III	DRR544816	0.216	*B. paranthracis*
BCER6	2008	August	177	13	47	9	11	68	12	10	IV	DRR544817	0.264	*B. cereus sensu stricto*
BCER7	2009	July	3284	122	8	8	11	9	12	85	IV	DRR544818	0.210	*B. cereus sensu stricto*
BCER8	2009	November	1351	254	30	277	37	44	199	5	III	DRR544819	0.163	*B. tropicus*
BCER9	2011	August	26	3	2	31	5	16	3	4	III	DRR544820	0.179	*B. paranthracis*
BCER10	2011	August	3285	3	2	59	56	71	83	98	III	DRR544821	0.216	*B. paranthracis*
BCER11	2011	October	73	13	8	9	14	9	12	31	IV	DRR544822	0.166	*B. cereus sensu stricto*
BCER13	2006	August	3286	34	5	32	1	18	47	24	III	DRR544823	0.149	*B. paranthracis*
BCER14	2006	September	3287	44	1	238	327	18	33	6	III	DRR544824	0.166	*B. paranthracis*
BCER15	2006	September	3288	247	1	12	109	55	102	210	III	DRR544825	0.258	*B. paranthracis*
BCER16	2007	August	3289	19	2	21	17	114	3	120	III	DRR544826	0.155	*B. paranthracis*
BCER17	2007	October	3290	13	8	9	11	68	13	85	IV	DRR544827	0.211	*B. cereus sensu stricto*
BCER18	2008	May	3291	19	39	21	5	114	3	120	III	DRR544828	0.211	*B. paranthracis*
BCER19	2008	May	177	13	47	9	11	68	12	10	IV	DRR544829	0.165	*B. cereus sensu stricto*
BCER20	2008	June	3292	122	8	218	11	9	12	10	IV	DRR544830	0.158	*B. cereus sensu stricto*
BCER21	2008	July	3293	34	1	71	370	33	33	6	III	DRR544831	0.171	*B. paranthracis*
BCER22	2008	September	3291	19	39	21	5	114	3	120	III	DRR544832	0.203	*B. paranthracis*
BCER23	2010	August	3292	122	8	218	11	9	12	10	IV	DRR544833	0.163	*B. cereus sensu stricto*
BCER24	2010	September	3294	13	8	8	11	11	12	85	IV	DRR544834	0.218	*B. cereus sensu stricto*
BCER26	2013	August	427	122	8	8	11	9	12	10	IV	DRR544835	0.172	*B. cereus sensu stricto*
BCER27	2014	December	26	3	2	31	5	16	3	4	III	DRR544836	0.175	*B. paranthracis*
BCER28	2015	June	3295	249	8	9	14	9	12	31	IV	DRR544837	0.263	*B. cereus sensu stricto*
BCER29	2017	January	26	3	2	31	5	16	3	4	III	DRR544838	0.185	*B. paranthracis*
BCER30	2017	December	3293	122	8	218	11	9	12	10	IV	DRR544839	0.175	*B. cereus sensu stricto*

^*a*^ MLST, multilocus sequence typing; *B., Bacillus*.

**Fig 3:**
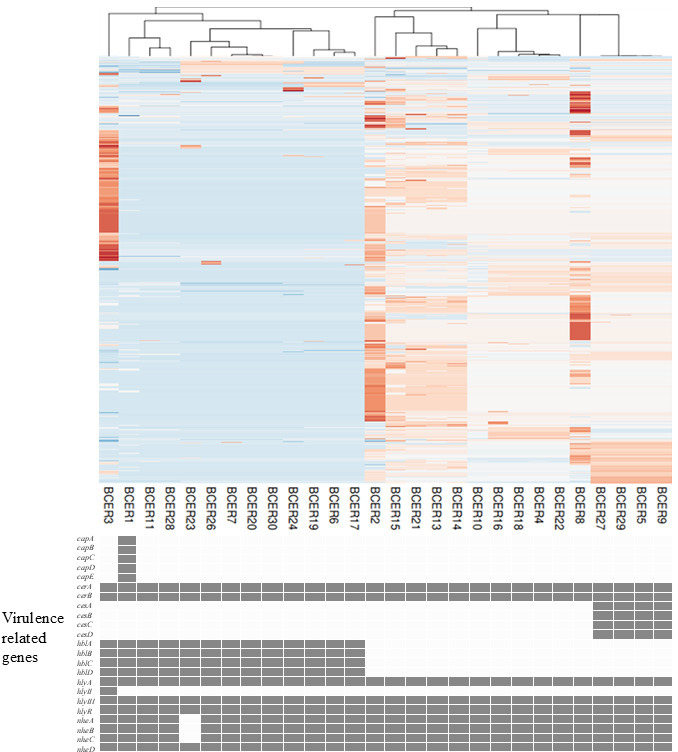
SNP profiles and virulence gene distribution of *B. cereus* isolates from bloodstream infections The upper panel shows a heatmap of 592 selected SNPs for the 28 *B. cereus* isolates based on whole-genome sequencing data. Each column represents an isolate, and each row represents a specific SNP position. The colors indicate different nucleotides at each SNP position: blue, red, white, and pink represent the four possible bases (A, T, G, and C, respectively). The lower panel displays the presence (gray boxes) or absence (white boxes) of virulence-related genes in each isolate. Virulence genes are grouped into categories including capsule genes (*capA–E*), cereulide synthetase genes (*cesA–D*), hemolysin BL genes (*hblA–D*), nonhemolytic enterotoxin genes (*nheA–C*), and other virulence-associated genes (*hlyA, hlyII, hlyIII,* and *plcR*).

Four cereulide synthetase gene (*cesABCD*)-containing isolates (BCER5, BCER9, BCER27, and BCER29) formed an independent branch in *panC* clade IV according to core-genome SNP typing (Fig. 2 and 3). Despite their close genetic relatedness, these isolates presented substantial genomic differences. Specifically, they differed by 18, 27, and 40 SNPs, respectively. Furthermore, these isolates did not share common routes of infection or isolation dates.

**Fig 4:**
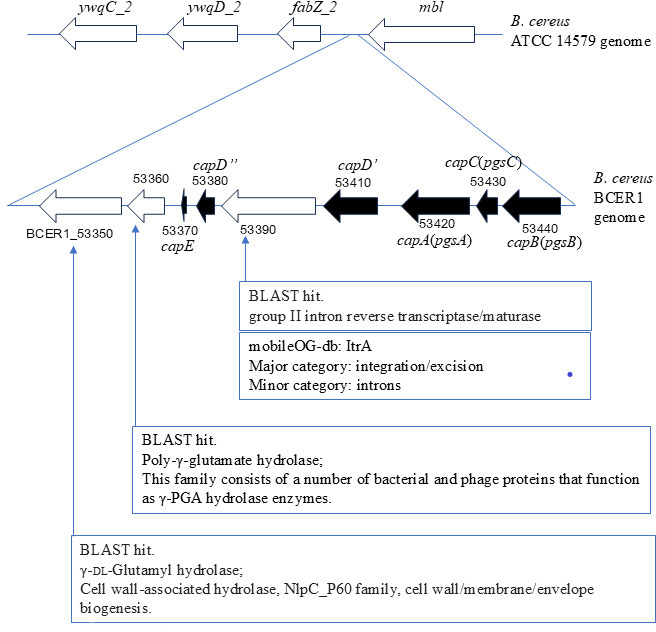
Genomic organization of the capsule gene operon in *B. cereus* BCER1 compared with the reference strain ATCC14579. The upper panel shows a section of the *B. cereus* ATCC14579 reference genome containing the *ywqC_2*, *ywqD_2*, *fabZ_2*, and *mbl* genes. The lower panel displays the corresponding region in the *B. cereus* BCER1 genome, where the capsule gene operon is inserted. Locus tags of BCER1_53350 to 54440 are marked. Arrows represent genes and their orientation. Black arrows indicate the capsule genes (*capB* (*pgsB*), *capC*(*pgsC*), *capA*(*pgsA*), *capD′*, *capD″*, and *capE*). White arrows represent other genes in this region, and BLAST hit annotations are provided for three genes adjacent to the capsule operon: group II intron reverse transcriptase/maturase, also classified as an integration/excision element (ItrA) in the mobileOG-db, and poly-γ-glutamate hydrolase enzyme and γ-dl-glutamyl hydrolase associated with cell wall/membrane/envelope biogenesis.

All 13 *panC* clade III isolates harbored *hbl* operon genes (Fig. 2 and 3). Only one isolate (BCER1) possessed the capsular operon gene (15), which was inserted into the chromosome between phage-related genes (Fig. 4).

## DISCUSSION

As reported previously, most *B. cereus* BSIs are caused by venous catheter-related infections (10). In the present study, no significant differences were found among the infection routes of biofilm-forming strains; however, catheter-related infection could still be a potential route for BSI. Most isolates were isolated in the summer, which is consistent with reports that *B. cereus* outbreaks are more common in the summer (21).

Our study revealed high genetic diversity among *B. cereus* group isolates causing BSIs. The isolates belonged to three *panC* clades, with a predominance of clades III and IV. This distribution is consistent with our previous findings on *B. cereus* group isolates from various sources in Japan (14). Interestingly, we did not identify any ST1420 isolates, which contrasts with earlier reports of the prevalence of ST1420 in nosocomial *B. cereus* infections in Japan (9). This absence could suggest geographical or temporal variations in strain distribution, but our limited sample size may have affected our ability to detect this sequence type.

All *ces* operon-positive isolates were genetically similar and assigned to ST26, which aligns with our previous findings on emetic *B. cereus* isolates (14). Notably, we identified one isolate possessing the complete capsule gene operon (*capBCADE*), a virulence factor associated with anthrax (15). The presence of this operon in a BSI isolate is concerning because capsulated bacilli can evade host immune responses more effectively (22).

Biofilm formation was observed for half of the catheter-related isolates. However, the limited sample size (*n* = 28) precluded establishing statistically significant associations between genetic characteristics, virulence factors, and clinical outcomes.

Despite these limitations, our study provides valuable insights into the genetic diversity and virulence potential of *B. cereus* group isolates causing BSIs in a Japanese hospital setting. The absence of previously reported prevalent sequence types, such as ST1420 (9), suggests potential shifts in strain distribution, although this requires confirmation in larger studies.

These results highlight the complex epidemiology of *B. cereus* BSIs and emphasize the need for continuous molecular surveillance. Our findings have important implications for infection control strategies in hospital settings, underscoring the necessity of tailored approaches to prevent and manage *B. cereus* infections. Future research should focus on larger, longer-term studies to elucidate virulence mechanisms, transmission patterns, and their impact on clinical outcomes. This approach will be crucial for developing more effective prevention strategies and improving the management of *B. cereus* infections in healthcare settings.

## Data Availability

The raw reads of *Bacillus cereus* group strains BCER2 to 11, 13 to 24, 26 to 30 have been deposited in DDBJ/GenBank under the accession number DRR544813 to DRR544839.

## SUPPLEMENTAL MATERIAL

Table S1 spectrum.02407-24-s0001.xlsx The consensus length, total read count, and average coverage of virulence gene identification using CLC genomics workbench for each isolate are shown.

ASM does not own the copyrights to Supplemental Material that may be linked to, or accessed through, an article. The authors have granted ASM a non-exclusive, world-wide license to publish the Supplemental Material files. Please contact the corresponding author directly for reuse.
