# Erratum for Okutani et al., "Genetic diversity and virulence of *Bacillus cereus* group isolates from bloodstream infections"

**DOI:** 10.1128/spectrum.02937-25

**Published:** 2025-10-10

**Authors:** Akiko Okutani, Shu Okugawa, Fumie Fujimoto, Mahoko Ikeda, Takeya Tsutsumi, Kyoji Moriya, Ken Maeda

## ERRATUM

Volume 13, no. 3, e02407-24, 2025, https://doi.org/10.1128/spectrum.02407-24. Page 9, Acknowledgments: “(grant number 24fk0108653)” should read “(grant number 24fk0108683).”

